# Is local autogenous morselized bone harvested from decompression through a posterior-transforaminal approach sufficient for single-level interbody fusion in the lower lumbar spine?

**DOI:** 10.1186/s12891-023-06131-4

**Published:** 2023-01-06

**Authors:** Jin Yang, Yong Yang, Gaoju Wang, Shuang Xu, Guangzhou Li, Shuai Zhang, Chaohua Yang, Song Wang, Qing Wang

**Affiliations:** 1 Department of Orthopedics Surgery, Affiliated Hospital of Southwest Medical University, 25 Taiping Road, Luzhou, 646000 Sichuan China; 2 Department of Orthopedics Surgery, Chengdu Yumei Hospital, 269 Xiajiancao Road, Chengdu, 610051 Sichuan China

**Keywords:** Volume of local bone harvesting, Local bone graft, Autogenous bone graft, Interbody fusion, Lumbar spine

## Abstract

**Background:**

To determine the volume and applicability of local autogenous morselized bone (LAMB) harvested and used during posterior-transforaminal lumbar interbody fusion (P-TLIF) in the lower lumbar spine.

**Methods:**

Clinical and radiographic data of 147 patients (87 males) undergoing P-TLIF from January 2017 to December 2019 for lumbar degenerative diseases were retrospectively analyzed. Computed tomography was used to assess the fusion status (at 6 months, 1 year, and the last follow-up postoperatively), restored disc height, graft fusion area and volume, and the minimum required bone volume (MRBV). Clinical outcomes of P-TLIF were assessed using the Oswestry Disability Index (ODI) and visual analog scale (VAS) for low back pain (LBP) and leg pain (LP).

**Results:**

The mean follow-up period was 28.4 ± 4.49 months. The patient’s age and diagnosis were correlated to the volume and weight of LAMB (mean volume and weight: 3.50 ± 0.45 mL and 3.88 ± 0.47 g, respectively). The ratio of actual fusion area to the total disc endplate and the ratio of actual fusion volume to the total volume of the disc space were > 40%. MRBV ranged from 1.83 ± 0.48 cm^3^ to 2.97 ± 0.68 cm^3^. The proportion of grade 4 or 5 fusions increased from 60.6% at 6 months to 96.6% at the last follow-up. The ODI, VAS-LP, and VAS-LBP scores significantly improved after surgery and remained unchanged during the follow-up.

**Conclusion:**

When combined with a cage, the volume of LAMB harvested from decompression through the unilateral approach at a single-level is sufficient to achieve a solid interbody fusion in the lower lumbar spine with excellent clinical and radiographic outcomes.

## Background

Spinal fusion is a commonly performed surgical procedure to treat various spinal morbidities, such as trauma, degenerative disc diseases, deformity, tumor, and infective pathologies [1, 2], of which, the majority are performed on the lumbar spine [3]. Solid fusion achieved using an excellent grafting material is the key to long-term spinal stability, as well as satisfactory clinical outcomes and quality of life [4, 5].

The ideal bone graft material should have inherent osteogenic, osteoinductive, and osteoconductive properties in addition to good mechanical strength [6]. An autologous iliac crest bone graft (ICBG) is considered the gold standard in terms of the aforementioned properties [4]; however, its use is limited due to the associated donor site pain, a limited supply of bone graft, and the need for additional surgical invasion [5, 6]. Therefore, alternative grafting materials, such as allografts, ceramics, demineralized bone matrix, and recombinant human bone morphogenetic proteins 2 and 7 (rhBMP-2 and 7) have been used in the clinical practice [7–9]. Although some of these materials are highly effective, their widespread use is hampered by certain limitations, such as the absence of osteogenic properties, the transmission risk for blood-borne pathogens, adverse or fatal events, and tremendous medical costs [7–10].

Recent studies have reported successful fusion in posterior lumbar interbody fusion and tansforaminal lumbar interbody fusion achieved using local bone grafts (LBG) derived from laminectomies, with fusion rates comparable to those using ICBG [11–14]. This may allow the patient to avert the need for an additional ICBG or other grafting materials while achieving the required solid fusion. However, to the best of our knowledge, no study has quantified the volume of local bone harvested from decompression through the unilateral approach and the amount of bone required for a single-level interbody fusion. Therefore, the present study aimed to quantify the volume of the local autogenous morselized bone (LAMB) harvested and the amount of bone used, to explore its applicability and clinical outcomes in posterior-transforaminal lumbar interbody fusion (P-TLIF) in the lower lumbar spine.

## Materials and methods

### Study population

We retrospectively analyzed patients undergoing P-TLIF for single-level lumbar degenerative diseases at our department between January 2017 and December 2019. In this study, the lower lumbar spine was defined as from L4 to S1. Ethical approval for the study was obtained from the Medical Ethics Review Committee of our hospital (Approval No: KY2022082).

### Inclusion and exclusion criteria

Patients with complete follow-up data who underwent P-TLIF for the following etiologies were included in this study: (1) single-level lumbar degenerative disease as confirmed by imaging characteristics and clinical symptoms, and decompressed unilaterally; (2) lumbar spinal stenosis (LSS), involving the lateral recess, foraminal stenosis, and central stenosis (Schizas grades B and C) [15, 16]; (3) lumbar segmental instability; (4) discogenic lower back pain (LBP) with a score > 5 measured using a visual analog scale (VAS) and confirmed by discography; (5) lumbar spondylolisthesis (Meryerding grade I and II), including degenerative or isthmic types; (6) lumbar disc herniation –highly migrated herniation, > 50% loss of disc height, and having chronic LBP (VAS > 5); (7) after more than 3 months of failed conservative treatment.

Patients with extreme LSS (Schizas grades D) [16]; a history of lumbar spinal surgery; osteoporosis (≤ − 2.5 standard deviation [SD] measured using dual-energy X-ray bone mineral density scan); or any other pathologies such as metabolic bone disorders, infections, or tumors were excluded from this study.

### Surgical intervention

**Fig. 1:**
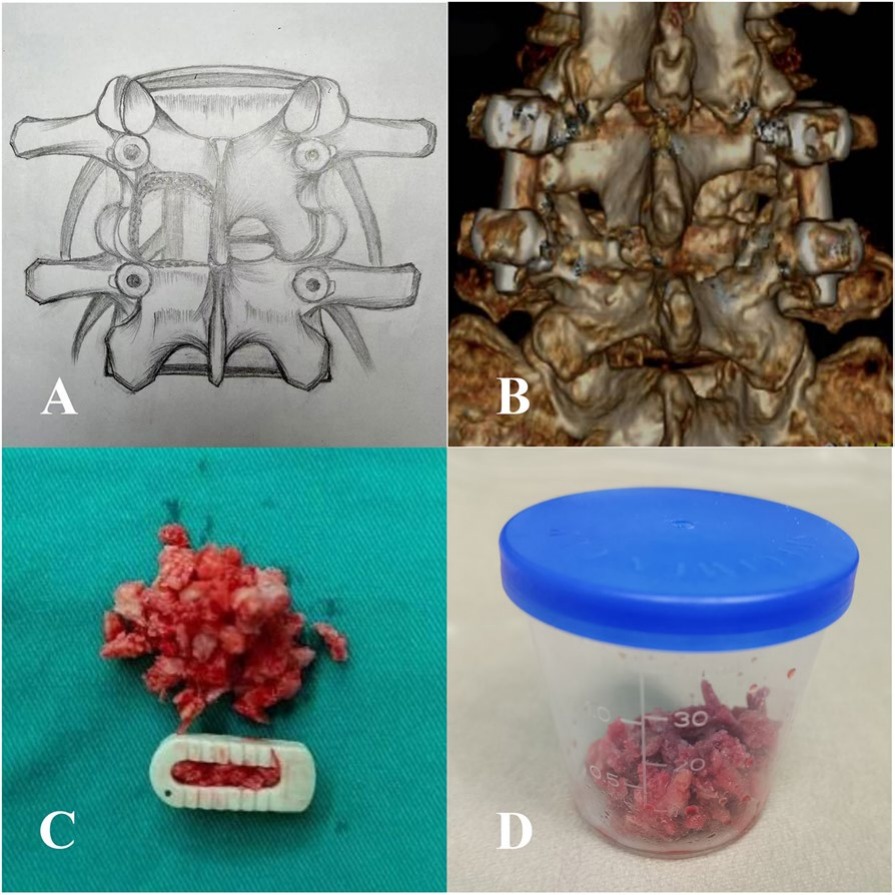
**A** The Schema indicates the extent of bony resection; **B** The extent of bony resection based on the three-dimensional reconstruction of computer tomography after surgery; **C** A cage filled with local autogenous morselized bone and the remaining bone; **D** A 40 mL sputum/specimen catch-cup containing bone for measurement

The surgical technique comprised a P-TLIF performed via a midline approach. After instrumentation, the lower facet and inferior two-thirds of the lamina of the upper vertebra, the upper one-fourths to one-thirds of the lamina and the medial half of the upper facet of the lower vertebra on the symptomatic side were removed to perform decompression (Fig. 1A and B). The LAMB harvested from the decompression was collected. The spinous process and interspinous ligaments remained intact. A nanohydroxyapatite/polyamide-66 cage (HA/PA66, Sichuan Guona Tech Co., Chengdu, China) was filled with LAMB (Fig. 1C). After achieving satisfactory decompression and thorough preparation of the graft bed, the remaining LAMB was inserted into the anterior part of the disc space and tamped gently. Next, the cage was placed into the disc space. Moe’s fusion technique [17] was used for the contralateral facet joint.

### Measurement of LAMB

All soft tissues were cleared from the harvested LAMB to ensure good bony fusion and cut of the appropriate size. The bone was put into a 40-mL sputum/specimen catch-cup (Fig. 1D) and weighed on a scale. Next, the cup was filled with normal saline, and the bone volume was calculated by subtracting the total volume of saline used from 40 mL. As the calculation of bone volume was based on the volume of saline used, we defined the volumetric ratio of the bone as equal to that of the saline. The bone was prepared by wrapping it with a piece of wet gauze until the graft was needed. The entire bone was used only for lumbar interbody fusion for each patient.

### Clinical evaluation

Clinical outcomes were assessed using the Oswestry Disability Index (ODI) and VAS scores for LBP and leg pain (LP) evaluated preoperatively and postoperatively (at 7 days, 6 months, 1 year after surgery, and on the last follow-up) by two independent reviewers (spinal surgeons).

### Radiographic evaluation

All radiographic parameters were evaluated by three independent reviewers (including two spinal surgeons and one senior radiologist). Any disagreements regarding non-quantitative parameters between the reviewers were resolved by reassessment and discussion. X-ray of the lumbar spine–lateral views including neutral, flexion and extension images, were used to evaluate the fusion segmental height, segmental stability, and the cage sinking condition. In this study, the segmental height was defined as the distance between the midpoint of the upper and lower endplates of the upper and lower fixed vertebrae. A change in the distance of > 3 mm was considered to be indicative of cage sinking.

### Fusion area and fusion status

Thin slice (1 mm) computed tomography (CT) scans and multi-planar reconstructions were used to assess the fusion condition. All measurements were conducted on a CT Advanced Workstation 4.4 (GE, USA). The graft fusion area was evaluated based on the pre and postoperative CT scans. The metal artifact reduction technique was used in post-operation CT scan after instrumented spinal surgery. The restored disc height, graft fusion area, and graft fusion volume, including results of the preoperative plan and the actual surgery, were measured and calculated. All parameters of the preoperative plan were calculated based on a 30% area of the target fusion disc [18]. Additionally, these parameters were compared using a stratification analysis according to different disc heights and different levels. The minimum required bone volume (MRBV) was calculated by subtracting the volume of the cage used from the planned graft fusion volume. The volume parameters of the cage of different model numbers were provided by Sichuan Guona Tech Co. Fusion status was assessed using the Brantigan classification [19] at 6 months, 1 year, and the last follow-up postoperatively. Successful fusion was defined as 4th and 5th fusion grades.

**Fig. 2:**
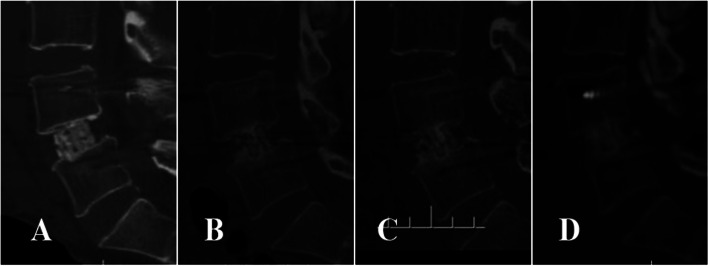
A series of post-operative computed tomography (CT) images of a 52-year-old female patient with L4–5 spinal stenosis reflecting the fusion progress over time. **A** Post-operative CT scan showing the fusion area; **B** the fusion grade of 3 at postoperative 6 months; **C** fusion grade of 4 at postoperative 12 months; and (**D**) fusion grade of 5 at the last follow-up

A representative case is presented in Fig. 2.

### Statistical analysis

All statistical analyses were conducted using SPSS (version 24.0, IBM Corp., Armonk, NY, USA). The normality of the data was tested using the Shapiro-Wilk test. Normally distributed continuous data were expressed as mean and SD and compared using the Student’s t-test. Between-group comparisons were made using a 1-way or repeated-measures ANOVA, followed by a pairwise comparison. The Chi-square (χ2) or Fisher’s exact tests were used to compare the frequencies of categorical data. A *p*-value of < 0.05 was considered statistically significant.

## Results

### Patient characteristics

We included 158 patients undergoing P-TLIF at our hospital during the study period, of which 11 were lost to follow-up (10 were lost due to a change in the contact details or address, 1 was lost due to death caused by a heart attack) and these patients with incomplete data were excluded from the study. The remaining 147 patients (males: *n* = 87, 59.1%; females: *n* = 60, 40.9%) were included in the final analysis. The demographic characteristics of these 147 patients are presented in Table 1. The mean operative time for all patients was 128.07 ± 18.93 minutes and the mean intraoperative blood loss was 248.7 ± 107.31 mL. On average, the hospital stay after surgery was 7.21 ± 0.94 days. The mean follow-up period was 28.41 ± 4.49 months.

**Table 1:** Demographic characteristics of 147 Patients

	Mean ± SD (range) or n (%)
Age (years)	59.76 ± 8.02 (44–79)
Sex male (%)	87 (59.1)
Body weight (kg)	60.22 ± 10.76 (45–90)
Height (cm)	167.27 ± 6.94 (149–183)
BMI (Body Mass Index)	21.47 ± 3.18 (15.9–29)
Duration of symptoms (months)	51.19 ± 38.16 (4–120)
Level involved	
L4–5	86 (58.6)
L5-S1	61 (41.4)
Sides left (%)	65 (44.2)
Diagnosis	
Lumbar spinal stenosis	63 (42.9)
Spondylolisthesis	40 (27.2)
Discogenic lower back pain	21 (14.3)
Lumbar segmental instability	13 (8.8)
Lumbar disc herniation	10 (6.8)
Comorbidities	
Hypertension	16 (10.8)
Diabetes	26 (17.7)
Chronic pulmonary disease	5 (3.4)
Smoking	35 (23.8)
Other surgery history	13 (8.8)
Surgical time (min)	128.07 ± 18.93 (100–170)
Intra-operative blood loss (ml)	248.78 ± 107.31 (110–700)
Hospital stay after surgery (d)	7.21 ± 0.94 (6–10)
Follow-up (month)	28.41 ± 4.49 (24–52)

**Fig. 3:**
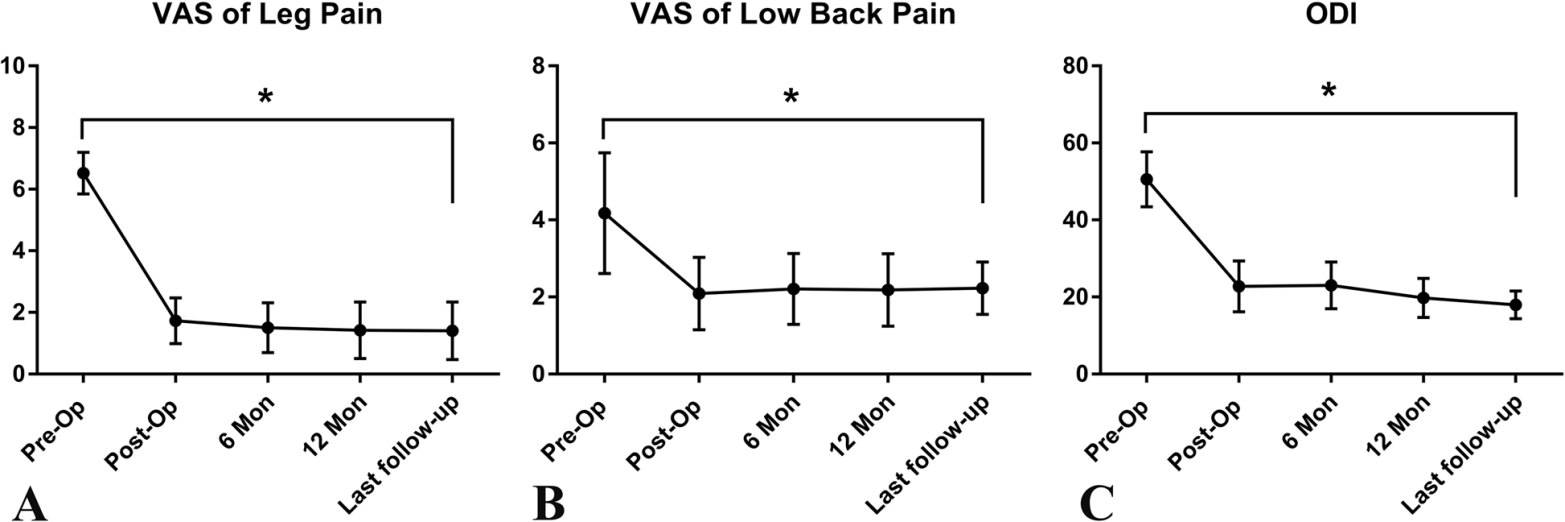
The improvement in visual analog scale score for low back pain (**A**) and leg pain (**B**), and the improvement in Oswestry Disability Index (**C**) after surgery along with the change in each outcome over time. *statistical significance between the preoperative and any postoperative value

The VAS score for LP decreased from 6.52 ± 0.68 preoperatively to 1.73 ± 0.74 postoperatively and 1.40 ± 0.94 at the last follow-up (*p* = 0.00). Likewise, the VAS score for LBP changed from 4.18 ± 1.57 preoperatively to 2.09 ± 0.94 postoperatively and 2.23 ± 0.68 at the last follow-up (*p* = 0.00). The ODI values decreased from 50.54 ± 7.17 preoperatively to 22.78 ± 6.62 postoperatively and 17.95 ± 3.62 at the last follow-up (*p* = 0.00) (Fig. 3).

### Measurement of LAMB

The mean volume and weight of the LAMB obtained from decompression was 3.50 ± 0.45 mL and 3.88 ± 0.47 g, respectively. Stratification analysis showed that the patient’s age and diagnosis were related to the volume and weight of LAMB (*p* < 0.05), i.e., the elder the patient, the more LAMB could be harvested. The maximum amount of bone was harvested from patients who underwent surgery for spondylolisthesis (3.62 ± 0.43 mL, 4.04 ± 0.44 g). Sex, body mass index (BMI), pathological levels, and side were not associated with LAMB volume and weight (*p* > 0.05) (Table 2).

**Table 2:** Local autogenous morselized bone obtained from decompression and related stratification analysis

	N	Bone Weight (g)	*P* value	Bone Volume (cm^3^)	*P* value
Local autogenous morselized bone	147	3.88 ± 0.47		3.50 ± 0.45	
Age (years)					
≤ 50	27	3.67 ± 0.42	0.028	3.34 ± 0.38	0.015
51–69	92	3.91 ± 0.45		3.51 ± 0.45	
≥ 70	28	3.98 ± 0.55		3.64 ± 0.50	
Sex					
Male	87	3.83 ± 0.45	0.116	3.45 ± 0.42	0.112
Female	60	3.95 ± 0.50		3.57 ± 0.49	
BMI (Body Mass Index)					
< 23.9	111	3.87 ± 0.48	0.752	3.49 ± 0.46	0.575
≥ 24	36	3.90 ± 0.47		3.54 ± 0.44	
Level involved					
L4–5	86	3.93 ± 0.50	0.145	3.53 ± 0.49	0.370
L5-S1	61	3.81 ± 0.42		3.46 ± 0.39	
Sides					
Left	65	3.81 ± 0.50	0.934	3.50 ± 0.47	0.951
Right	82	3.88 ± 0.46		3.50 ± 0.44	
Diagnosis					
Lumbar spinal stenosis	63	3.92 ± 0.46	0.010	3.59 ± 0.40	0.001
Spondylolisthesis	40	4.04 ± 0.44		3.62 ± 0.43	
Discogenic lower back pain	21	3.68 ± 0.44		3.20 ± 0.42	
Lumbar segmental instability	13	3.62 ± 0.51		3.42 ± 0.52	
Lumbar disc herniation	10	3.76 ± 0.48		3.24 ± 0.47	

### Evaluation of bone graft and fusion status

Regarding the comparison between the preoperative plan and the surgical results, we found that the graft fusion area and volume were statistically significant (*p* < 0.05), whereas the restored disc height was not significant (Table 3). Also, the stratification analysis revealed that the planned graft fusion area and volume were statistically correlated with actual results in terms of the differences in disc height and levels (Table 4). The ratio of actual fusion area to the total disc endplate ranged from 40.5 to 42%; whereas, the ratio of actual fusion volume to the total disc space volume ranged from 40.6 to 46.1%. The volumes and weights of bone harvested from decompression under the different disc heights and at different levels are presented in Table 4. MRBV ranged from 1.83 ± 0.48 cm^3^ to 2.97 ± 0.68 cm^3^ (Table 4).

**Table 3:** The comparison between the preoperative plan and actual results of the surgery for 147 patients

	Preoperative plan	Actual result	*P* value	*t* value
Restored disc height (mm)	10.81 ± 1.46	10.90 ± 1.43	0.628	−0.485
Graft fusion area (mm^2^)	423.60 ± 62.77	576.85 ± 105.47	0.000	−15.139
Graft fusion volume (cm^3^)	4.58 ± 0.95	6.29 ± 1.41	0.000	−12.196

The bone graft area and bone graft volume in the preoperative plan were calculated based on the bone graft area of 30% endplate of the target segment

**Table 4:** Comparison between the planned required bone volume and the actual results of different disc heights achieved using the bone harvested from decompression

Disc Height (mm)	8 (*n* = 18)		10 (*n* = 54)		12 (*n* = 72)	
Level	L4–5 (*n* = 8)	L5-S1 (*n* = 10)	L4–5 (*n* = 33)	L5-S1 (*n* = 21)	L4–5 (*n* = 44)	L5-S1 (*n* = 28)
Planned graft fusion area (mm^2^)	460.80 ± 52.94	415.05 ± 61.32	415.81 ± 71.20	400.89 ± 57.73	431.40 ± 55.12	431.19 ± 70.13
Actual graft fusion area (mm^2^)	645.48 ± 83.62	565.13 ± 131.34	562.18 ± 111.17	540.72 ± 102.53	594.37 ± 100.05	586.97 ± 100.10
*P* Value	0.000	0.004	0.000	0.000	0.000	0.000
The ratio of actual fusion area to the total disc endplate (%)	42.0	40.8	40.6	40.5	41.3	40.8
Planed graft fusion volume (cm^3^)	3.68 ± 0.44	3.31 ± 0.48	4.15 ± 0.72	4.01 ± 0.57	5.18 ± 0.68	5.17 ± 0.85
Actual graft fusion volume (cm^3^)	5.65 ± 0.96	4.62 ± 1.11	5.74 ± 1.30	5.45 ± 0.98	7.11 ± 1.21	6.99 ± 1.13
*P* Value	0.000	0.003	0.000	0.000	0.000	0.000
The ratio of actual fusion volume to the total volume of the disc space (%)	46.1	41.9	41.5	40.8	41.2	40.6
Bone volume harvested from decompression (cm^3^)	3.53 ± 0.57	3.64 ± 0.37	3.49 ± 0.40	3.40 ± 0.40	3.54 ± 0.53	3.45 ± 0.39
Bone weight harvested from decompression (g)	3.99 ± 0.56	4.02 ± 0.39	3.89 ± 0.47	3.73 ± 0.43	3.93 ± 0.52	3.81 ± 0.43
Minimum required bone volume (cm^3^)	2.20 ± 0.44	1.83 ± 0.48	2.31 ± 0.72	2.17 ± 0.57	2.97 ± 0.68	2.96 ± 0.85

The minimum required bone volume (MRBV) was calculated as: MRBV = planed graft fusion volume - the volume of the cage; Fusion area included the area of bone graft and the cage; Fusion volume included the volume of bone graft and the cage; Planed fusion area, Planed Fusion volume, MRBV were all calculated based on the bone graft area of 30% endplate of the target segment

**Fig. 4:**
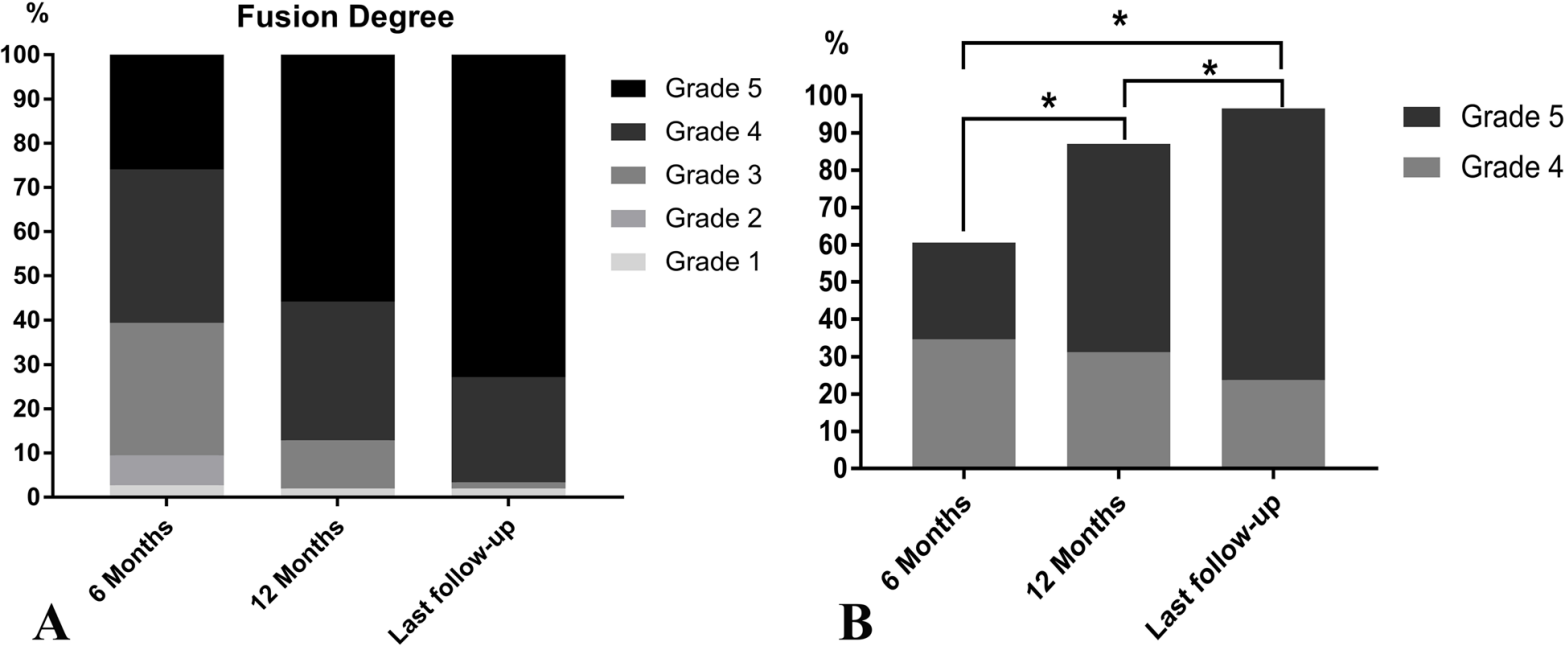
**A** The degree of fusion progress and **B** the comparison of fusion rates between different time points after surgery

Approximately 39.4% (*n* = 58) of the patients had grades 1, 2, or 3 fusion, whereas, the majority (*n* = 89, 60.6%) of the patients had grade 4 or 5 fusion at 6 months postoperatively. Notably, the number of grade 4 or 5 fusions increased to 87.1% (*n* = 128) at 1 year and 96.6% (*n* = 142) at the last follow-up. The difference in the fusion rates of grades 4 and 5 between the three time points (6 months, 1 year, and the last follow-up) was statistically significant (*p* < 0.05) (Fig. 4).

### Complications

An intraoperative dural tear occurred in three patients (2%); and nerve root edema was noted in 4 patients (2.7%); however, these nerve injuries did not cause any severe complications. Grade 1 fusions were developed in three patients (2%), including two at the L5-S1 level and one at L4–5, due to pseudoarthrosis, graft bone absorption, cage sinking, or screw breakage. Two of them underwent revision surgery, while the third refused and was under follow-up. Two patients (1.4%) with cage sinking and one (0.7%) with pedicle screw displacement without neurologic deficit at 6 months postoperatively underwent conservative treatments and eventually achieved grade 4 fusion. Adjacent segment disc degeneration was found in five asymptomatic patients (3.4%) who did not receive any additional treatment.

## Discussion

To the best of our knowledge, this is the first study to quantify the volume of local bone harvested from decompression through a unilateral approach and the amount of bone required for interbody fusion at a single level of the lower lumbar spine. Excellent fusion status and satisfactory clinical outcomes were observed after using LAMB during P-TLIF for an average of 28 months of follow-up.

### The extent of bony resection and LAMB

We found that the volume of LAMB harvested from unilateral decompression (mean volume and weight: 3.50 ± 0.45 cm^3^ and 3.88 ± 0.47 g, respectively) was sufficient for a single-level interbody fusion in the lower lumbar spine. The mean volume of bone harvested in the present study was significantly less than that of bone harvested in a previous study (mean: 25 cc) [20]; this difference may be attributed to the extent of bony resection. In the previous study using posterolateral fusion, half of the cranial and caudal spinous processes, the entire lamina, and the bilateral facets were removed; in contrast, we did not perform such a wide bony resection and decompression. Furthermore, in contrast to the previous study [20], we did not include patients with extreme LSS [16] because these patients always need a total laminectomy to achieve complete posterior decompression. In most patients, unilateral direct decompression combined with indirect decompression achieved by restoring disc height using a cage could be sufficient to achieve satisfactory decompression. Additionally, our procedure was primarily aimed at preserving the integrity of the spinal structures and maintaining stability to minimize the risk of LBP and adjacent disc degeneration after surgery.

Currently, lumbar interbody fusion is one of the mainstream fusion methods to achieve superior clinical outcomes and higher fusion rates. Theoretically, the volume of required bone graft for interbody fusion is smaller than that of posterolateral fusion [20], which was also confirmed by our results. A previous study reported that, from a biomechanical perspective, the area of intervertebral fusion should be greater than 30% [18]; therefore, we defined the 30% of the target disc area as the minimal bone graft area in the preoperative plan. Furthermore, our results showed the actual graft fusion area was significantly larger than that in the preoperative plan (*p* = 0.00). Moreover, the stratification analysis for different disc heights and spinal levels also showed similar results. Furthermore, the ratio of the actual fusion area to the total disc endplate ranged from 40.50 to 42%. These results suggest that the volume of LAMB and corresponding graft fusion area exceed the need for fusion based on 30% fusion area of the target disc.

Age was positively associated with the volume of LAMB harvested. Spondylolisthesis and LSS had the largest volume of LAMB. We hypothesized that this additional bone volume was associated with the degree of spinal degeneration and secondary hyperplasia of bone. The more the age, the more severe is the lumbar degeneration. Additionally, lumbar spondylolisthesis and LSS always cause more severe degenerative changes than discogenic LBP, lumbar segmental instability, and lumbar disc herniation. Moreover, severe arthritis, hyperplasia of the facets, and thickened lamina were more frequently seen in these two pathologies. Therefore, these changes increased the volume of resultant LAMB. Other patient characteristics, such as sex, BMI, sides, and spinal levels were not related to the LAMB volume, which is unlike a previous study that reported a trend of greater local bone graft volumes in men [20].

### Fusion status

Previous studies had proved the ability of LBG for lumbar interbody fusion with a fusion rate of 94.5 to 100% for at least 2 years of follow-up. In the present study, the final fusion rate of grade 4 or 5 was achieved in 96.6% of patients, which was comparable with previous studies [12, 13, 21, 22]. Our results also concur with the findings of studies using other bone grafts and biologics to achieve comparable fusion rates: 89.6% using bioactive glass-local autograft mixtures, 91.6% using autograft, 95.8% or 98.3% using iliac crest, and 94.1% using rhBMP-2 and local bone graft at 1-year follow-up [9, 12, 13, 22]. These results establish not only the applicability of LAMB for interbody fusion in the lower lumbar spine but also the fact that the volume of LAMB harvested from the decompression is sufficient to achieve fusion at a single level.

Moreover, the CT-based fusion grade and fusion rate increased significantly over time. The proportion of cases estimated as grade 4 or 5 increased from 60.6% at 6 months to 87.1% at 1 year and 96.6% at the last follow-up (*p* < 0.05). Therefore, we agreed that spinal fusion is an ongoing process and radiological nonunion after 1 year should not be regarded as definitive failure [23]; accordingly, these patients should be followed up diligently. The complication rate associated with fusion and instrumentations in this study was only 7.5% which is much better than previous studies that used LBG or using other bone grafting materials [9, 12, 13, 22].

### Clinical outcomes

Clinically, the patients’ condition continued to improve and maintained the improvements up to an average of 28 months in terms of ODI and VAS for LBP and LP. We believe that these excellent clinical outcomes can be attributed to the high fusion rate, minimal surgical invasion during the procedure, and maintenance of the maximum integrity of the posterior ligament complex. However, the relationship between the radiographic and clinical outcomes of lumbar fusion surgery remains controversial [23–27]. Several studies have reported that higher fusion rates did not significantly improve the clinical outcomes, whereas others have demonstrated the long-term clinical benefits of solid fusion over pseudarthrosis [23, 25–27].

## Limitations

This study has several limitations. First, only single-level P-TLIF cases with unilateral decompression in the lower lumbar spine were studied. Whether this procedure will be applicable for the treatment of multilevel P-TLIF or upper lumbar P-TLIF is yet to be established. Second, the time point of the last follow-up was different due to patient compliance. Third, the bone volume measurement putting the bone in a cup with saline may lose osteoprogenitor cells. Finally, the retrospective nature of the study may have introduced selection bias.

## Conclusion

This novel study reported that when combined with a cage, the volume of LAMB harvested from decompression through the unilateral approach at a single-level is sufficient to achieve solid interbody fusion in the lower lumbar spine with excellent clinical and radiographic outcomes.

## Data Availability

The datasets in the current study are available from the corresponding author on reasonable request.

## Abbreviations

BMI: Body mass index
CT: Computed tomography
ICBG: Iliac crest bone graft
LBG: Local bone grafting
LAMB: Local autogenous morselized bone
LBP: Low back pain
LP: Leg pain
LSS: Lumbar spinal stenosis
MRBV: Minimum required bone volume
ODI: Oswestry disability index
P-TLIF: Posterior transforaminal lumbar interbody fusion
rhBMP: Recombinant human bone morphogenetic protein
VAS: Visual analog scale

## References

[1] Nyström B, Weber H, Schillberg B, Taube A (2017). Symptoms and signs possibly indicating segmental, discogenic pain. A fusion study with 18 years of follow-up. Scand J Pain.

[2] Machado GC, Ferreira PH, Yoo RI, Harris IA, Pinheiro MB, Koes BW (2016). Surgical options for lumbar spinal stenosis. Cochrane Database Syst Rev.

[3] Boden SD (2002). Overview of the biology of lumbar spine fusion and principles for selecting a bone graft substitute. Spine (Phila Pa 1976).

[4] Shen F, Samartzis D, An H (2005). Cell technologies for spinal fusion. Spine (Phila Pa 1976).

[5] Myeroff C, Archdeacon M (2011). Autogenous bone graft: donor sites and techniques. J Bone Joint Surg Am.

[6] VonderHoeh NH, Voelker A, Heyde CE (2017). Results of lumbar spondylodeses using different bone grafting materials after transforaminal lumbar interbody fusion (TLIF). Eur Spine J.

[7] Feng JT, Yang XG, Wang F, He X, Hu YC (2020). Efficacy and safety of bone substitutes in lumbar spinal fusion: a systematic review and network meta-analysis of randomized controlled trials. Eur Spine J.

[8] Li GB, Li PQ, Chen Q, Thu HE, Hussain Z (2019). Current updates on bone grafting biomaterials and recombinant human growth factors implanted biotherapy for spinal fusion: a review of human clinical studies. Curr Drug Deliv.

[9] Letchuman V, Ampie L, Choy W, DiDomenico JD, Syed HR, Buchholz AL (2021). Bone grafting and biologics for spinal fusion in the pediatric population: current understanding and future perspective. Neurosurg Focus.

[10] Food and Drug Administration. FDA Public Health Notification: Life-threatening complications associated with recombinant human bone morphogenetic protein in cervical spine fusion. Available at http://www.fda.gov/MedicalDevices/Safety/AlertsandNotices/PublicHealthNotiications/ucm062000.htm.

[11] Hashimoto T, Oha F, Shigenobu K, Kanayama M, Harada M, Ohkoshi Y (2001). Mid-term clinical results of Graf stabilization for lumbar degenerative pathologies: a minimum 2-year follow-up. Spine J.

[12] Adams CL, Ogden K, Robertson IK, Broadhurst S, Edis D (2014). Effectiveness and safety of recombinant human bone morphogenetic protein-2 versus local bone graft in primary lumbar interbody fusions. Spine (Phila Pa 1976).

[13] Ito Z, Matsuyama Y, Sakai Y, Imagama S, Wakao N, Ando K (2010). Bone union rate with autologous iliac bone versus local bone graft in posterior lumbar interbody fusion. Spine (Phila Pa 1976).

[14] Kim DH, Jeong ST, Lee SS (2009). Posterior lumbar interbody fusion using a unilateral single cage and a local morselized bone graft in the degenerative lumbar spine. Clin. Orthop Surg.

[15] Wang Y, Dou Q, Yang J, Zhang LF, Yan YQ, Peng ZY (2018). Percutaneous endoscopic lumbar decompression for lumbar lateral spinal canal stenosis: classification of lateral region of lumbar spinal canal and surgical approaches. World Neurosurg.

[16] Schizas C, Theumann N, Burn A, Tansey R, Wardlaw D, Smith FW (2010). Qualitative grading of severity of lumbar spinal stenosis based on the morphology of the dural sac on magnetic resonance images. Spine (Phila Pa 1976).

[17] Moe JH (1957). The management of paralytic scoliosis. South Med J.

[18] Closkey RF, Parsons JR, Lee CK, Blacksin MF, Zimmerman MC (1993). Mechanics of interbody spinal fusion. Analysis of critical bone graft area. Spine (Phila Pa 1976).

[19] Brantigan JW, Steffee AD (1993). A carbon fiber implant to aid interbody lumbar fusion. Two-years clinical results in the fi rst 26 patients. Spine (Phila Pa 1976).

[20] Carragee EJ, Comer GC, Smith MW (2011). Local bone graft harvesting and volumes in posterolateral lumbar fusion: a technical report. Spine J.

[21] Miura Y, Imagama S, Yoda M, Mitsuguchi H, Kachi H (2003). Is local bone viable as a source of bone graft in posterior lumbar interbody fusion?. Spine (Phila Pa 1976).

[22] Ito Z, Imagama S, Kanemura T, Hachiya Y, Miura Y, Kamiya M (2013). Bone union rate with autologous iliac bone versus local bone graft in posterior lumbar interbody fusion (PLIF): a multicenter study. Eur Spine J.

[23] Lehr AM, Oner FC, Delawi D, Stellato RK, Hoebink EA, Kempen DHR (2020). Increasing fusion rate between 1 and 2 years after instrumented posterolateral spinal fusion and the role of bone grafting. Spine (Phila Pa 1976).

[24] Dhall SS, Choudhri TF, Eck JC, Watters C, Dailey AT, Resnick DK (2014). Guideline update for the performance of fusion procedures for degenerative disease of the lumbar spine. Part 5: correlation between radiographic outcome and function. J Neurosurg Spine.

[25] Kornblum MB, Fischgrund JS, Herkowitz HN, Abraham DA, Berkower DL, Ditkoff JS (2004). Degenerative lumbar spondylolisthesis with spinal stenosis: a prospective long term study comparing fusion and pseudarthrosis. Spine (Phila Pa 1976).

[26] Tsutsumimoto T, Shimogata M, Yoshimura Y, Misawa H (2008). Union versus nonunion after posterolateral lumbar fusion: a comparison of long-term surgical outcomes in patients with degenerative lumbar spondylolisthesis. Eur Spine J.

[27] Djurasovic M, Glassman SD, Dimar JR, Bratcher KR, Carreon LY (2011). Does fusion status correlate with patient outcomes in lumbar spinal fusion?. Spine (Phila Pa 1976).

